# An approach to assessing subsea pipeline-associated mercury release into the North Sea and its potential environmental and human health impact

**DOI:** 10.1098/rsos.230943

**Published:** 2024-03-13

**Authors:** Rebecca von Hellfeld, Astley Hastings

**Affiliations:** ^1^ School of Biological Sciences, University of Aberdeen, 23 St Machar Drive, Aberdeen AB24 3UL, UK; ^2^ National Decommissioning Centre, Main Street, Newburgh AB41 6AA, UK

**Keywords:** contaminant modelling, ecopath with ecosim, food standards for mercury, estimated weekly intake, hazard quotient

## Abstract

Mercury is a naturally occurring heavy metal that has also been associated with anthropogenic sources such as cement production or hydrocarbon extraction. Mercury is a contaminant of concern as it can have a significant negative impact on organismal health when ingested. In aquatic environments, it bioaccumulates up the foodweb, where it then has the potential to impact human health. With the offshore hydrocarbon platforms in the North Sea nearing decommissioning, they must be assessed as a potential source for the environmental release of mercury. International treaties govern the handling of materials placed in the ocean. Studies have assessed the ecologic and economic benefits of (partial) *in situ* abandonment of the infrastructure as artificial reefs. This can be applied to pipelines after substantial cleaning to remove mercury accumulation from the inner surface. This work outlines the application of an approach to modelling marine mercury bioaccumulation for decommissioning scenarios in the North Sea. Here, *in situ* decommissioning of cleaned pipelines was unlikely to have a negative impact on the North Sea food web or human health. However, significant knowledge gaps have been determined, which must be addressed before all negative impacts on ecosystems and organismal health can be excluded.

## 1. Introduction

The North Sea is a very biodiverse ecosystem [1], with strong primary producers and a stable benthic community [2]. Furthermore, the North Sea is of global importance for seabirds and marine mammals [1]. In addition to its high biodiversity, the North Sea is one of the world’s most important fishing grounds, with herring and mackerel being among the most commercially relevant species [3]. The North Sea also holds a total of 590 oil and gas platforms [4], of which >470 platforms are expected to be decommissioned over the coming decades in the UK sector alone. This includes around 5000 extraction wells and ~45 000 km subsea pipelines [5]. This can be completed through a variety of methods ranging from complete removal of all installed infrastructure to *in situ* abandonment of the cleaned pipeline and jacket for artificial reef creation (known as ‘rigs-to-reefs’) [6]. Such options have been implemented in the Gulf of Mexico [7] and are being considered for the North Sea [8]. Here, the proposed decommissioning plan should aim to minimize the impact on the marine environment, as well as limit human risks in line with current regulations. These include the ‘United Nations Convention on the Law of the Sea’ (UNCLOS) [9], the ‘Convention on the Prevention of Marine Pollution by dumping of wasted and other materials 1972 (London Convention)’ and the London Protocol [10], as well as the Oslo and Paris Conventions (OSPAR) decision 98/3 on the disposal of disused offshore installations [11]. In addition, legislations such as the Marine Strategy Framework 46 Directive 2008/56/EU [12] and 2011/92/EU [13] must be considered, which state that each ‘project’ must assess all direct and indirect impacts on biota, the environment, material assets and cultural heritage. This provides guidance for considerations to be taken in the assessments conducted in decommissioning plans, determining scenarios with minimal impact of the planned operations on the ecosystem and surrounding environment, as well as limiting human risk and economic costs [14].

It has been argued that the *in situ* abandonment of offshore infrastructure could lead to an increase in biodiversity or biomass of the associated fish assemblage through the formation of artificial reefs [8]. However, little focus is currently placed on the effects of residual contaminants potentially associated with the infrastructure, even after cleaning [15–17]. One contaminant of concern is mercury, a naturally occurring heavy metal that is extracted alongside the hydrocarbon from offshore reservoirs [18,19]. Mercury is known to associate with the internal pipeline surfaces owing to rapid changes in temperature and pressure [20,21]. Mercury speciates based on surrounding parameters and the availability of other compounds [22]. In most aquatic ecosystems, 95% of mercury is sediment-associated, bound to organic matter and mineral phases [23]. Common insoluble mercury species found in marine ecosystems include cinnabar (HgS), calomel (Hg_2_Cl_2_), mercuric oxide (HgO) and elemental mercury (Hg^0^). Soluble mercury chloride complexes (HgCl_2_, HgCl_3_^−^, HgCl_4_^2+^) and dissolved or particulate organic matter complexes (DOM-Hg and POM-Hg, respectively) are also present [24]. Although it is known that parameters such as temperature, pH, salinity, pore water sulphide, organic matter and sediment redox potential influence speciation [18], the complexity of the marine environment increases the difficulty in accurately determining the fate of mercury in the ocean and its accumulation in marine biota [19]. Mercury also speciates in oil and gas production systems based on their physical and chemical properties. The water-soluble Hg^0^ forms more stable complexes with chloride or sulphur ions [25]. Studies showed that Hg^0^ adsorbs to the inner steel surface of pipelines [26–28]. Mercury also adsorbs on corrosion products formed within the pipelines. Here, the speciation process is based on environmental parameters, as well as the corrosion scale make-up [18]. Although sulphur-bound species are initially unavailable for biota uptake, at low sulphide concentrations, such as those found in oxic seawater, the dissolved fraction of mercury increases [29]. For a detailed analysis of the parameters influencing pipeline-bound mercury speciation, refer to [18]. While pipeline-cleaning methods such as pigging and chemical cleaning exist [30,31], studies have estimated that even cleaned pipelines may still contain up to 98 mg total mercury per kg pipeline (herein mg kg^-1^) [32]. The same study further determined a sediment concentration of 260 µg total mercury per kg sediment (herein µg kg^-1^) at the pipeline. This concentration decreased to 10 µg kg^-1^ at 125 m from the pipeline. In comparison, the concentration given by OSPAR for pristine or remote sites is 50 µg kg^-1^, while the accepted concentration assessment is 70 µg kg^-1^ for marine sediments [33]. However, considering that published data for cleaning previously active subsea pipelines is scarce, and we currently lack the technology for measuring *in situ* mercury contamination in pipelines, it cannot be excluded that parts of to-be-abandoned pipelines may hold higher mercury concentrations than laboratory studies indicated [18].

For organismal health, the most important speciation process is the methylation of mercury into organic methylmercury, a known neurotoxicant [34]. While freshwater sediments are assumed to be the main source of mercury methylation [35], processes driving marine methylation are less well understood. Studies suggest that the formation here is driven by the mixed layer [36]. On average, 10% of the total mercury in the water column is methylmercury. This increases to 15% in phytoplankton, 30% in zooplankton and 95% in fishes and higher trophic-level organisms, owing to its bioaccumulation and biomagnification potential [19]. Once ingested, methylmercury has a long retention time, thus leading to its bioaccumulation within an organism, as well as its biomagnification in food webs [37]. Approximately 90% of ingested methylmercury is thought to be absorbed through the gastrointestinal tract, as its strong affinity for L-cysteine allows it to cross cell membranes and enter the blood stream [38,39]. The L-type neutral amino acid carrier transport 1 system then enables its transport into the central nervous system [40]. Methylmercury also covalently bonds with sulfhydryl group [41], which leads to enzyme inhibition and may be the cause of its neurotoxicity [42]. To account for this toxicity, a tolerable weekly intake (TWI) for methylmercury has been developed [43]. This governs the maximum permissible body burden from dietary intake that would not likely induce negative health effects in humans. The current TWI of 1.3 µg methylmercury per kg body weight [44] (herein referred to as µg kg bw^-1^) was based on epidemiological studies and is considered protective of the developmental neurotoxic effects of methylmercury ingestion in foetuses and children and should be followed by women of childbearing age or those who are pregnant. However, special focus should be placed on considering changes in dietary mercury exposure for future generations.

### 1.2. Aims and objectives

The research aims to apply a previously outlined method [45] for mercury food web bioaccumulation modelling to the North Sea considering different exposure scenarios. An existing eEcopath with an eEcosim model of the North Sea was used [46], with varied mercury influx into the model environment, based on different decommissioning scenarios for the North Sea. The modelled data were validated against current mercury concentrations in different organisms. This approach allowed for the determination of potential data gaps for mercury accumulation in the North Sea, as well as knowledge gaps that require addressing to make this method suitable for environmental impact assessments in the future. Furthermore, the economic implications were calculated in terms of revenue lost owing to increased mercury accumulation beyond the current food standards (FS) for mercury. Moreover, the estimated weekly intake of methylmercury (EWI_MeHg_) was calculated for men and women, in general, as well as children and pregnant women, comparing the obtained EWI_MeHg_ to the current TWI. In addition, the hazard quotient (HQ) for these groups was derived to assess the overall risk posed to each.

## 2. Material and methods

To better understand bioaccumulation of mercury in the North Sea marine food web, a previously outlined *in silico* food web modelling approach [45] for Ecopath with Ecosim (EwE, V.6 [47]) and its contaminant tracking tool ‘Ecotracer’ [48] was used. The model was calibrated using literature-derived data for environmental mercury concentrations in the North Sea, and biota uptake rates. The results obtained in the present study were assessed against literature-derived mercury concentrations in biota samples from the North Sea. A previously developed food web model of the North Sea [46] was used to test the method, and different environmentally relevant exposure scenarios (ES) were determined. Data conversions were conducted using Microsoft Excel^®^, and the data visualization was done using the Tidyverse package for R or SigmaPlot (V.14.0, Systat Software Inc.) [49,50].

### 2.1. Food web modelling with Ecopath with Ecosim and Ecotracer

A detailed explanation of the underlying equations and functions of EwE can be found in Christensen *et al*. [51]. Briefly, EwE is a food web modelling programme based on determining the static, temporal or spatiotemporal mass balance of a modelled system. The underlying calculations and input values regarding parameters such as mortality, migration and predation, form the basis for the contaminant tracking tool ‘Ecotracer’. Ecotracer simulates the transport of a chosen contaminant through the food web, solving the contaminant dynamic equation simultaneously with the mass balance [52]. It allows for a varied contaminant influx over time and considers different decomposition/outflow pathways. A detailed description of the tool equations and assumptions was recently published [48]. Ecotracer has been used to track mercury and methylmercury in marine food webs in previous studies. This includes a theoretical study highlighting the suitability of the programme suite for environmental risk assessment for offshore decommissioning [45] and the impacts of climate change on the health risks to the Faroe Island population owing to their reliance on marine mammal meat [53].

### 2.2. Model calibration

**Figure 1.**
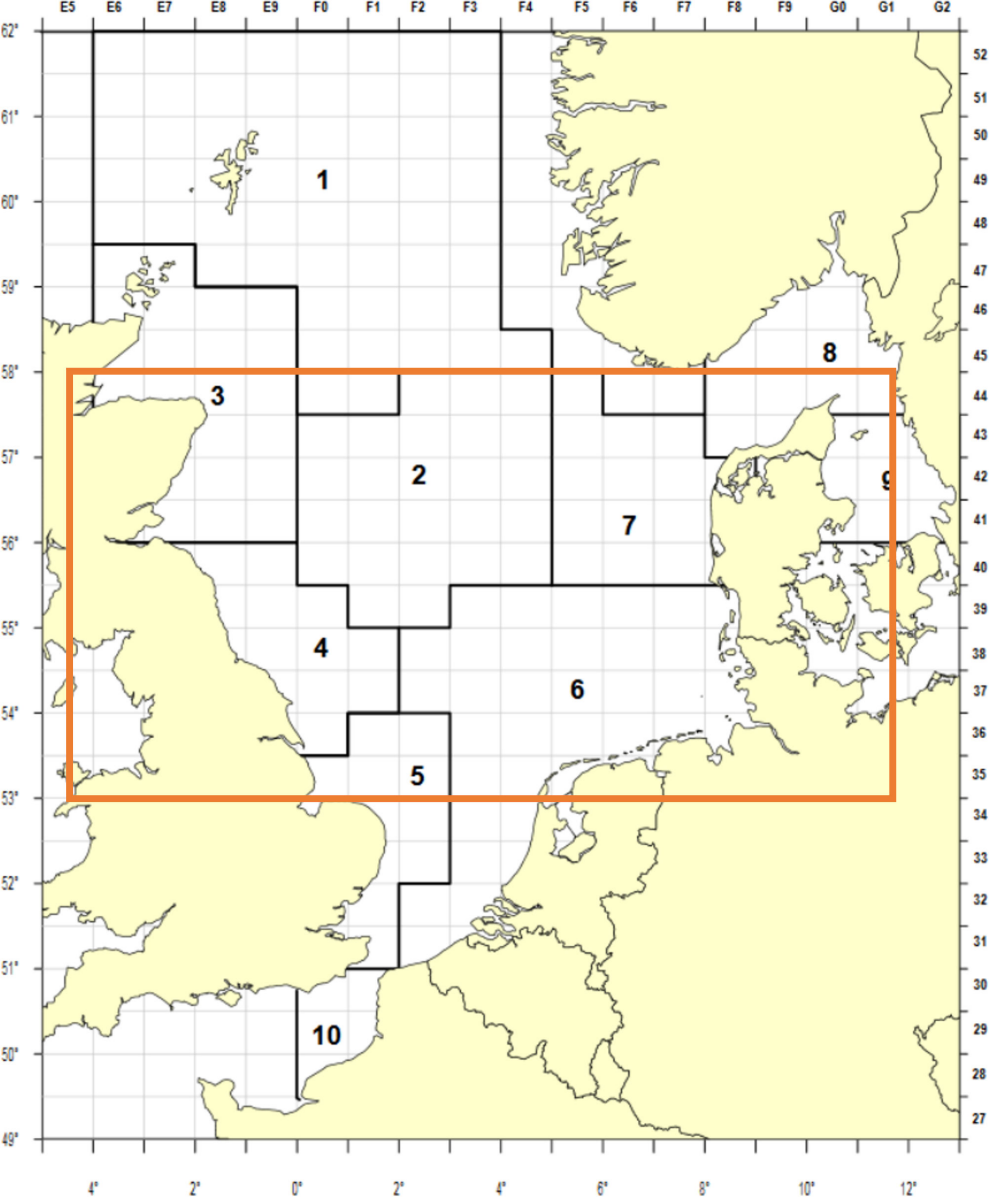
Map of the North Sea, with the International Council for the Exploration of the Sea (ICES) statistical rectangles. The orange rectangle highlights the area considered here. Adapted from ICES, 2018 [59].

In the present study, an existing and validated EwE model of the North Sea was used [46], covering the area outlined in figure 1. The model contains 29 functional groups, including the detritus, and a full species list and the EwE input parameters can be found in the electronic supplementary material, S1. To calibrate the model, an initial environmental concentration was derived from published data, and separate model runs were initiated to incorporate the environmental mercury into the different species. This was done following the method outlined in a recent publication on a hypothetical foodweb [45]. Direct mercury uptake was only derived for photosynthetic plankton groups, copepods and euphausiids, as the literature suggests most uptake for other marine organisms is diet-based [53–56]; see the electronic supplementary material, S2, for details. For the remaining functional groups, the direct uptake of mercury from seawater is considered negligible compared with the dietary uptake [57] and thus not computed. For this study, exposure to total mercury was modelled, using the uptake and retention time parameters to simulate methylmercury biota uptake. To this end, an excretion rate of 10% was modelled. This accounts for the uptake of non-accumulating mercury species, as well as the potential for some species to demethylate small amounts of mercury [58].

The initial environmental total mercury concentration used here is 0.57 µg l^-1^, based on the data from the International Council for the Exploration of the Sea (ICES) ‘Contaminants and biological effects of contaminants in water’ dataset [60]. This value is derived from a dataset encompassing almost 300 000 data points from 1979 to 2021 (see https://dome.ices.dk/views/ContaminantsBiota.aspx for a detailed review of quantification methods and study characteristics). This value is higher than other publications provided, as a large uncertainty pertains to values obtained pre-1958 owing to changes in measurement method. However, for the present work, a conservative background concentration was found suitable, as it aims to test a method and determine data gaps. Moreover, the model validation outlined below highlights that, even with this conservative environmental concentration, the species modelled accumulate less mercury than was measured in comparable species in the North Sea. A representation of the environmental mercury concentrations in the North Sea can be found in the electronic supplementary material, S3.

### 2.3. Model validation at equilibrium

Before determining the changes in bioaccumulation of mercury, the environmental mercury concentration and direct uptake rates for the selected groups were input into Ecotracer and the system was allowed to reach equilibrium. The resulting mercury burden for each species was converted into muscle tissue concentration and compared to mercury concentrations measured in biota samples. Based on literature findings, approximately 50% of total mercury accumulates in muscle tissue for most fish species [61], 15% for shrimp and 100% for benthos species [62–64], plankton [65,66] and detritus. Such values can be compared with the FS for mercury in foodstuffs, determining that 0.5 and 1 mg kg^-1^ muscle tissue are guideline values for low and high trophic level species, respectively [67]. These values indicate whether landed fish is eligible for sale commercially.

The initial mercury concentrations for each functional group derived from EwE were compared with literature-derived concentrations of North Sea species with the same trophic level. Only publications were considered where the species’ trophic level was discernible, muscle samples were analysed, it was evident whether the concentration was provided as wet or dry weight fraction, only fresh samples with an uninterrupted freezing were assessed and the work was published in English. To validate the modelled values, the model/observed (*M*/*O*) ratio was derived as follows [68]:


(2.1) $$M/O\;ratio=\frac{M}{O}$$

where O is the modelled and *O* is the mean observed mercury concentration. The closer to 1 the derived value is, the more closely the modelled value represents the observed value. The normalized mean bias (NMB), where *n* is the number of empirical studies generating the observed mean mercury concentration value [68]:


(2.2) $$NMB=\frac{\sum_{1}^{n}\left(M-O\right)}{\sum_{1}^{n}O}.$$

If the NMB ≥ 0, the model overpredicts the observation by a factor equivalent to the NMB + 1. For example, a derived factor of 1.2 shows an overestimation by the model of a factor 2.2, while a value of −0.2 highlights an underprediction by a factor of 1.2, for example [69]. The results of the validation can be found in the electronic supplementary material, S4. The implemented environmental mercury concentration may exceed the current concentration measured in North Sea marine samples, but when calibrating the model, current concentrations did not lead to comparable concentrations in modelled organisms with biota samples analysed. This indicates the suitability of the selected value to the food web model. Such discrepancies can be based on the limitations inherent in modelling studies, such as those discussed in this work. This publication intends to highlight the method and its current data and knowledge gaps, this deviation was accepted as suitable to the task.

### 2.4. Exposure scenarios

Once the model equilibrium was validated, four scenarios were derived, representing current environmental mercury concentrations (ES A), *in situ* decommissioning of some pre-cleaned pipelines based on current decommissioning proposals (ES B), and *in situ* decommissioning of all pipelines in the North Sea after cleaning (ES C and D); see the electronic supplementary material, S5 for actual values and details.

#### 2.4.1. Exposure scenario A: current environmental concentrations

ES A represents the current environmental total mercury concentration of 0.57 µg l^-1^ [60], as outlined above. To estimate the annual anthropogenic influx of total mercury into the North Sea, the ‘inputs of mercury, cadmium and lead via water and air to the Greater North Sea between 1990 and 2014’ dataset [70], provided by the Centre for Environment, Fisheries and Aquaculture Science (CEFAS) was used. The average influx from 2005 to 2014 of the CEFAS-provided dataset was used (0.01 t yr^-1^), providing a more conservative input value than an average of the entire dataset would provide [70]. For conversions into the model environment, a mean depth of 95 m and an area of 570 000 km^2^ was assumed, as these are the average depth and the overall area of the North Sea.

#### 2.4.2. Exposure scenario B: 317 km *in situ* decommissioned cleaned pipeline

ES B represents a scenario where all 317.12 km of pipeline currently abandoned in the North Sea and without a clear plan for removal [71] are accepted for *in situ* decommissioning after cleaning. From the publicly accessible data, the average pipeline is a schedule 60 with a 10″ diameter (outer diameter: 27.31 cm, wall thickness: 12.7 mm). The recent decommissioning report states that a cleaned pipeline is estimated to lead to a concentration of 260 µg kg^-1^ in the sediment directly surrounding the pipeline [32]. To model the potential release of a decommissioned pipeline, it was assumed that a pipeline released 260 µg kg^-1^, which equates to a total concentration of 0.07 t mercury in 317.12 km pipeline. The environmental mercury concentration and annual influx, as well as initial species concentration as stated in ES A were applied in ES B. No difference in the final accumulation of contaminants in the model was observed when comparing single-release events versus setwise release (data not shown) [45]. Thus, ES B assumes a single-release event after 50 years of 0.07 t yr^-1^, before returning to the CEFAS database-derived annual anthropogenic influx.

#### 2.4.3. Exposure scenario C: 45 000 km *in situ* decommissioned cleaned pipeline

ES C represents a release scenario where all 45 000 km pipelines currently in the North Sea are decommissioned *in situ* post cleaning. The assumptions and values of ES A apply, and, similar to ES B, at 50 years, an additional influx of 9.9 t mercury was added to the system, before returning to the previously used 0.000004 t yr^-1^, this would equate to a total concentration of 9.9 t mercury in 45 000 km cleaned pipeline.

#### 2.4.4. Exposure scenario D: 45 000 km *in situ* decommissioned pipelines cleaned to smelting regulations

ES D represents a release scenario where all 45 000 km pipeline currently in the North Sea are decommissioned *in situ* post cleaning. The assumptions and values of ES A apply, and the same calculations as for ES B and C apply. However, here, the pipelines are cleaned to the maximum threshold given by many smelting companies, which is 2 mg kg^-1^ steel. For 45 000 km pipeline, this would lead to an additional influx of 72.93 t mercury after 50 years before returning to the previously used 0.000004 t yr^-1^.

### 2.5. Model assumptions

ES B and C represent a concentration series as all underlying assumptions remain the same as outlined in ES A with the addition of the single release of additional mercury. While the above described method is the most suitable option for the present study, incremental release modelling may be more suitable for shorter timescale studies or instances where spatial modelling is considered. Further limitations include that it was assumed that all mercury entering the marine environment is available for uptake, and actual body burden data of North Sea species were used to compute uptake rates. This represents a very conservative (i.e. ‘worst case scenario’) example that may not be applicable to every ecosystem. Moreover, all applied simplifications follow the method initially outlined in von Hellfeld *et al*. [45]. To determine the partitioning of a contaminant between the water and sediment/particulate compartment, a partitioning coefficient (*K*_d_ value) can be employed [72]. However, these values must be derived for each environment and are highly dependent on the mercury species, as well as parameters such as organic matter content. To this end, the assumptions in the previous work [45] were accepted for the sake of testing the method for the North Sea. It was further assumed that no sediment mixing or dilution occurs post pipeline release, limiting the dispersion potential for mercury in the aqueous phase. A sediment compartment was not modelled. A closed model environment was assumed, with no migration of biota or import/export of aqueous mercury, owing to a lack of data. Although the model validation highlighted the fit of the model to current environmental mercury concentrations and accumulation, the change in marine organism muscle concentration in ES B and C may not be representative of future scenarios. The results presented here merely outline the final mercury concentration based on the outlined assumptions and estimates. Considering that mercury biomagnification will be highly sensitive to local environments and ecosystems, as well as to changes in local parameters and characteristics, these results should not be interpreted as being predictive of impacts from real mercury releases.

### 2.6. Estimated weekly intake of methylmercury and hazard quotient determination

To derive the EWI_MeHg_ for humans from consuming seafood with total mercury concentrations equivalent to those derived from the ES, previously described methods were followed [45]. Here, only fish landed in ICES ecoregion codes IIIa, IV (a–c) and VIId and VIIe were considered as ‘North Sea’ [73]. This approach determined that 79% of fish and 77% of shellfish in the United Kingdom originated from non-North Sea landings [74]. Moreover, all seafood imported into the UK was also considered for the determination of the EWI_MeHg_. As not enough data for background mercury concentrations in fish landed abroad or imported could be obtained, the concentration determined in ES A was applied to the imported proportion. For seafood originating from the North Sea, muscle concentrations determined through the model scenarios were applied.

To determine the EWI_MeHg_, the bioaccessibility of methylmercury during consumption (the fraction of tissue-bound methylmercury that is released from the food matrix during consumption and in a soluble form in the gastrointestinal tract, see the electronic supplementary material, S6) and uptake rates of methylmercury by human stomach epithelial cells (79% [75]) were calculated by adapting the following equation [45]:


(2.3) $$EWI_{MeHg}=\frac{AC\times F\times WI\times AB}{W}\times Frac.$$

Using the mercury muscle tissue concentration (AC, µg kg^-1^), the fraction represents bioaccessible methylmercury (F, %), the weekly fish or seafood intake (WI, kg), the methylmercury stomach epithelial cell absorption rate (AB, %), the body weight (W, kg) and the fraction of consumed seafood (Frac). The weekly seafood consumption was determined based on the National Health Service (NHS) recommendations for seafood consumption [76], and the average weekly consumed seafood was given by the National Diet and Nutrition Survey (NDNS) [77] (electronic supplementary material, S7).

To determine the impact of increased mercury concentration in fish, the weekly intake for each food group was determined by dividing the respective weekly intake value by the number of species in the group:


(2.4) $$foodgroup=\sum \frac{\frac{F\times W}{n_{group}}}{n_{group}}.$$

The results for all three seafood groups were then combined to provide the EWI_MeHg_ for total weekly seafood consumption.

The HQ for methylmercury (HQ_MeHg_) was calculated to determine the ratio between the potential exposure to a substance, and the level of exposure at which no adverse effect is anticipated [78]. An HQ ≤ 1 indicates that adverse effects are not likely to occur, while an HQ > 1 states whether, and by how much, an exposure dose (ED) exceeds the reference concentration (RfC) for dietary exposure. The HQ is calculated as outlined below [79]:


(2.5) $$HQ_{MeHg}=\frac{ED}{RfC}.$$

For this study, the RfC_MeHg_ of 0.0001 mg kg^-1^ d^-1^ (or 0.0007 mg kg^-1^ week^-1^) as defined by the United States Environmental Protection Agency (EPA) was applied [80].

## 3. Results

### 3.1. No exceedance of the food standards for mercury in seafood in any modelled scenario

**Figure 2.**
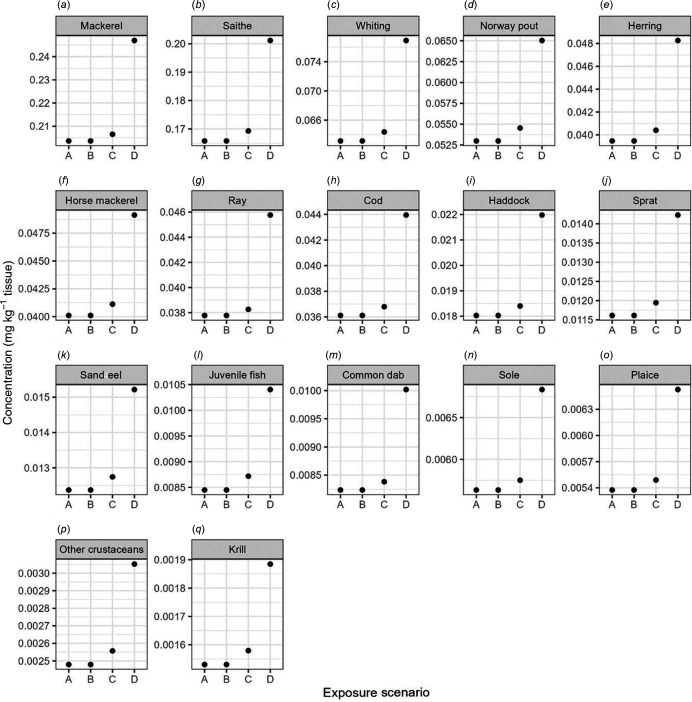
Final muscle tissue total mercury concentration (mg kg^-1^) in commercially relevant modelled North Sea species (*a*–*q*) (shown as circles) for the four exposure scenarios (A–D) as highlighted in the methodology. Total muscle concentrations are listed in the electronic supplementary material, S9.

The muscle tissue concentration in all species increased in line with an increase in influx of mercury into the model environment (figure 2). In ES A, representing an unchanged influx of mercury into the marine environment, none of the commercially relevant species were observed to exceed either of the FS for mercury. None of the observed species accumulated mercury in amounts that would lead to an exceedance of the FS for mercury in ES B to D. However, in ES D, mackerel and saithe were modelled to contain muscle tissue mercury concentrations above 0.2 mg kg^-1^. None of the modelled concentrations exceed the FS for mercury of 0.5 and 1 mg kg^-1^ for high and low trophic level organisms, respectively.

### 3.2. No exceedance of the estimated weekly intake of methylmercury and limited increase of the hazard quotient for all modelled scenarios

**Figure 3.**
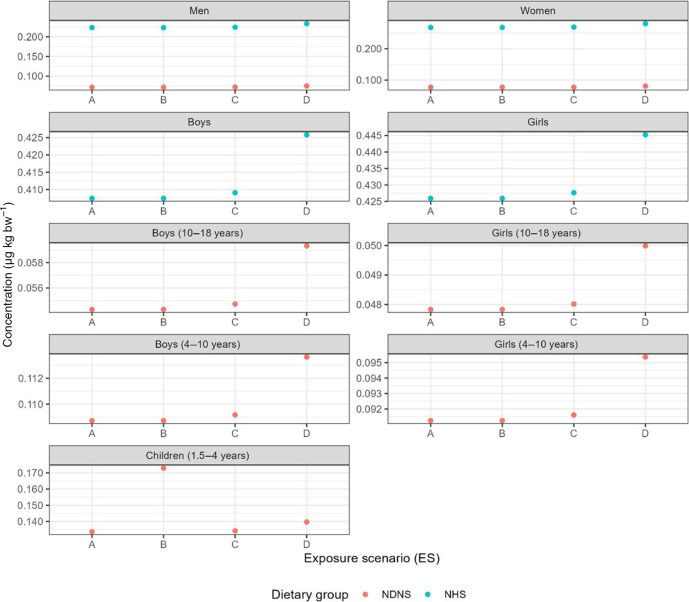
EWI_MeHg_(µg kg bw ^-1^) for the total weekly seafood intake derived for NHS recommendation of seafood consumption (teal) and the UK average weekly consumed seafood as given by the NDNS (coral) in the respective consumer group (figure labels); see the electronic supplementary material, S10 for more information.

**Figure 4.**
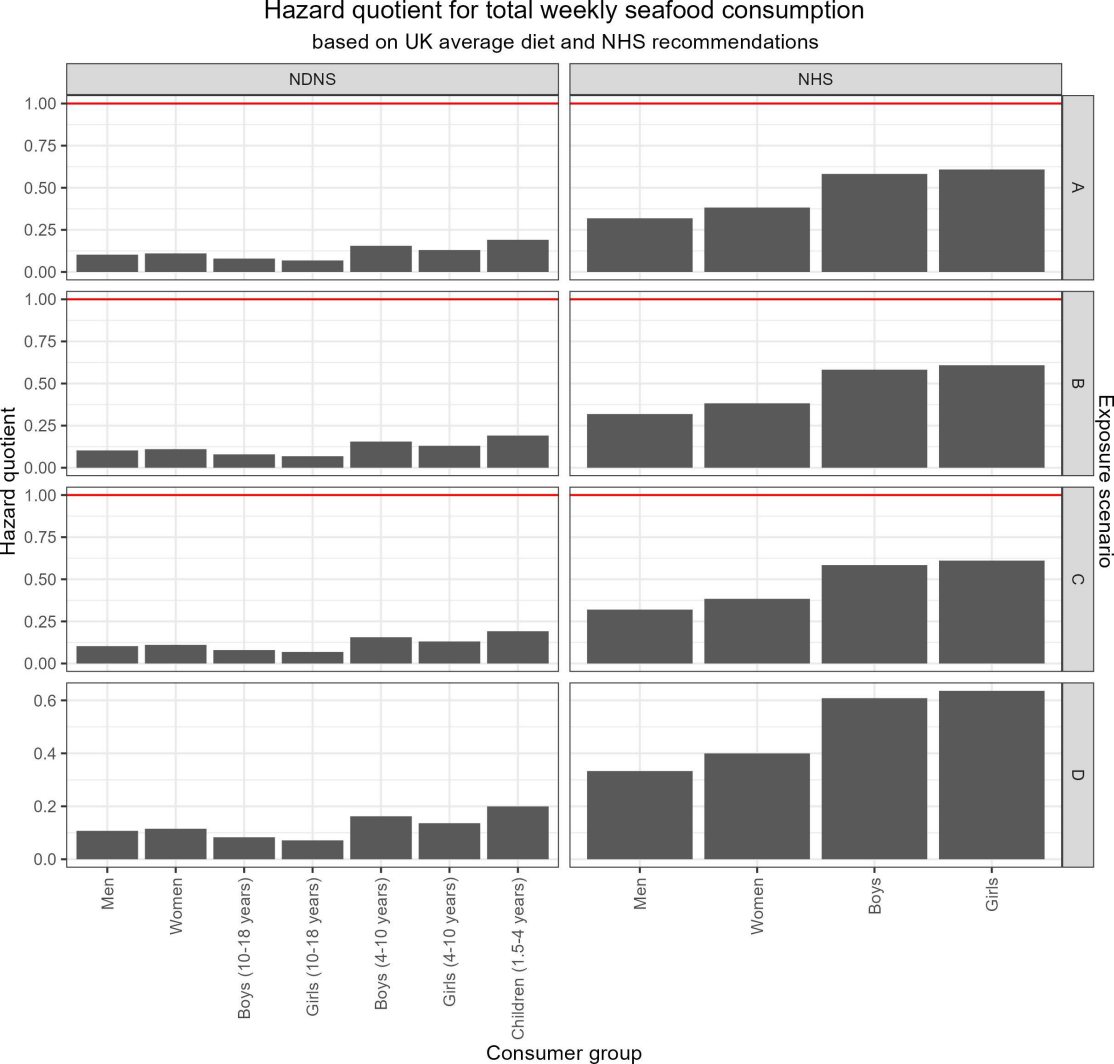
HQ for different consumer groups (as listed on the *x*-axis) and dietary intake (top labels for the UK average weekly seafood consumption according to the NDNS, and the NHS weekly recommendations) for all four exposure scenarios (vertical labels on the right). The red line indicates the HQ = 1 threshold; see the electronic supplementary material, S11 for more information.

In none of the modelled scenarios did the EWI_MeHg_ of total seafood consumption per week by any consumer group under either of the consumption rates exceed the current TWI of 1.3 µg kg bw^-1^ [44] (figure 3). Following the NHS dietary recommendations [76], all groups were modelled to take in more methylmercury per week, than when following the actual current seafood consumption according to the NDNS [77]. A similar trend was observed when determining the HQ for all consumer groups (figure 4).

## 4. Discussion

Overall, the data presented in this study have shown that no direct risk from *in situ* decommissioning of oil and gas pipelines in the North Sea is to be expected, under the outlined assumptions. Although increased concentrations of mercury in muscle tissue were modelled, the burden was not found to surpass the FS for mercury in seafood. Current North Sea biota mercury concentrations cited in the ICES database [81], as well as in the Marine Scotland database [82] and peer-reviewed publications [83,84], for example, highlighted that various species of interest exceed the FS for mercury. Although the presented data for all scenarios determined that modelled fish species did not exceed the FS for mercury (figure 2; electronic supplementary material, S9), the model validation process highlighted that the *M*/*O* and NMB values derived for the model were conservative to highly conservative for all but one species modelled (electronic supplementary material, S4). This suggests that although approaching biota concentrations, the mercury accumulation in many modelled species was still underestimated. Lastly, although increases in the EWI_MeHg_ and the HQ were computed, none of the respective threshold values were surpassed.

The modelled approach of the present work does not account for sub-lethal impacts on marine organisms, which may occur at modelled concentrations below threshold values such as the FS for mercury in seafood. To this end, the following discussion will provide a more detailed insight into the current understanding of the ecological and organismal health impacts of mercury overall. Although no immediate risk to human health was modelled, a deeper assessment of the potential implications for future communities is required to better understand the risk factors and implications. Considering the model limitations and considerations that will be discussed, risk and impact assessments should nonetheless be carried out for each new decommissioning plan.

### 4.1. Ecological and organismal impacts of sub-lethal mercury concentrations

Current approaches to food web contaminant accumulation rarely assess the deleterious effect such concentrations may have on the organisms at or below the modelled concentration. It is known that long-term exposure to sub-lethal concentrations of mercury can impact organismal health over time. Such impacts include developmental alterations and impaired larval predator avoidance and prey-capture abilities in mummichog embryos (*Fundulus heteroclitus*) exposed to <0.1 mg l^-1^ methylmercury [85–87]. The direct link between laboratory studies and environmental exposure is lacking. Publications indicating the mercury content in wild-caught fish are available [88,89], but in such cases, behavioural or developmental alterations are not recorded. Only a few publications examine whether environmental mercury exposure affects the development or behaviour of wild organisms [90,91]. Similarly, limited data are available on mercury and methylmercury toxicity of marine species, a shortcoming recently highlighted by Gissi *et al*. [19]. This highlights that although we are capable of modelling mercury in marine ecosystems and understanding the lethality in wild animals, we still lack the translation of what impact exposure to sub-lethal concentrations has outside of laboratory conditions. This is a vital gap that requires addressing, as current threshold values such as the FS for mercury are tailored to human health, rather than animal wellbeing.

### 4.2. Human health assessment: the global context

In the present study, no model scenario led to an EWI_MeHg_ exceeding the TWI of 1.3 µg kg bw^-1^ (figure 3). The TWI estimates the amount per unit body weight of a contaminant that can be ingested over time without risk of adverse effects. For methylmercury, the TWI takes into consideration that extended exposure will increase the health risks through its accumulating potential [44]. Contrary to the data presented here, a recent study highlighted that five of the assessed European countries (Belgium, Ireland, Italy, Portugal and Spain) had higher subgroups of the population that were potentially at risk of EWI_MeHg_ > 1.3 µg kg bw^-1^ TWI (table 1). With the overall conservative *M*/*O* values for the present study, an underestimation of the EWI_MeHg_ is plausible. Although overall comparable, future studies would benefit from a unified methodology for the determination of methylmercury intake in different groups of the population. Additionally, testing of both raw and processed samples would provide more insight into the potential EWI_MeHg_ for different processing methods and would allow a better understanding of a consumer group’s risk based on personal and regional preferences. Such an approach was successfully employed by Bradey *et al*. [92]. They also highlighted that the thus far accepted fraction of >95% of total mercury being methylmercury does not hold true for all aquatic species. More in-depth sample analysis is necessary to avoid an overestimation of risk from lower trophic level species (i.e. accumulating less mercury) in the future.

**Table 1.** Summary of EWI_MeHg_ from seafood from different European and international studies in comparison to the present study.

**Study, Region**	**EWI_MeHg_ (µg kg bw^-1^)**	**Additional information**	**Reference**
Present study, UK	> 0.5		Present study
FP7-ECsafeSEAFOOD-project, EU	>TWI	From hake, cod, seabream, sea bass and octopus	[93]
‘CALIPSO study’, France	1.3–1.6	Tuna, cod, ling, sole and hake were the main contributors to the methylmercury intake [94]	[44]
‘Seafood frequency consumption questionnaire’, Mediterranean Sea	0.12–6.11	For women of reproductive age	[95]
N/A, Spain	0.98–2.60	For vulnerable population groups (children, pregnant women and women of childbearing age)	[96]
N/A, Mallorquín swamp	5.3, 3.7 and 4.4	For children, women of childbearing age and the rest of the population, respectively	[97]
BfM MEAL, Germany	>TWI	From the consumption of tuna, herring, pollock and trout (smoked, canned in oil or brine, fried, pickled or in sauce)	[98]

Like the EWI_MeHg_, no modelled scenario was determined to lead to HQ >1 through seafood consumption (figure 4). The HQ is the ratio between the dietary exposure dose and the reference dose, which is based on a compound-specific benchmark concentration and an uncertainty factor to account for bioaccumulation and other toxic effects [78]. Although the derived HQ value does not provide a quantitative assessment of the potential risk, the larger the value, the higher the difference between the exposure concentration and the reference concentration and thus the greater the level of concern [79]. Various studies have assessed the HQ for methylmercury from seafood consumption (table 2). An exceedance of a HQ > 1 by certain consumer groups was not always concurrent with an exceedance of the accepted TWI [99]. Similarly, an exceedance of the FS for mercury by certain fish species was not always indicative of an increased HQ by consumer groups. Such findings highlight the need for a better understanding of risk factors to different consumer groups, as well as taking dietary preferences and cultural factors into account.

**Table 2.** Summary of EWI_MeHg_ from seafood from different European and international studies.

**Region**	**Finding**	**Reference**
Zhoushan, China	The HQ for children and adults exceeded 1 for total seafood and fish. This was found even when the EWI_MeHg_ did not exceed the TWI	[99]
Baja California Sur, Mexico	Not all species with muscle concentrations exceeding the FS for mercury also lead to a HQ > 1	[100]
Aracaju, Brazil	Six out of 13 analysed species exceeded the 0.5 mg kg^-1^ and two species exceeding the 1 mg kg^-1^ FS for mercury. Only two species induced a HQ > 1	[101]
Malaysia	HQ Between 1.5 and 2.7 for urban and coastal rural women of childbearing age, respectively	[102]
Jeddah, Saudi Arabia	Only one of the 13 samples fish species exceeded the 0.5 mg kg^-1^ FS for mercury. Seven species had a HQ > 1	[103]

### 4.3. The foodweb model: limitations and considerations

The modelled data presented here show that EwE can successfully be used to model mercury accumulation in a North Sea food web. Only one species accumulated more mercury than current biota samples indicate present in such species (electronic supplementary material, S4). This supports the future use of easy-to-use programmes such as EwE for comprehensive impacts and risk assessments for ecosystems. As modelling remains a simplification of real-world scenarios, limitations must be considered. For the present study, these limitations include: (i) the bioaccumulative nature of mercury in marine animals not being represented in EwE, (ii) the lack of knowledge of additional sources of mercury, (iii) the insufficient data on uptake rates and contaminant burden for model calibration, and (iv) the speciation of mercury in marine ecosystems.

When calibrating the model, an inflated environmental mercury concentration was used to achieve a biota accumulation that is representative of published data for different North Sea species. This may be addressed by including factors like bioaccumulation and biomagnification in the future EwE-Ecotracer version. To account for the increase in mercury with each trophic level, a highly conservative environmental mercury concentration was employed here. Such approaches can increase the data uncertainty at higher concentrations and should thus be addressed prior to the implementation on a larger scale. Such improvements could include the addition of factors to account for the biomagnification within the food web or different species. Although methods for the determination of bioaccumulation factors for contaminants exist, and data are available for food webs, these are often standalone [104] rather than an integrated variable in food web modelling.

The environmental mercury concentration in the North Sea and the annual influx were derived from publicly accessible databases [60,70]. Additional influx sources may not have been monitored, leading to an underestimation of the North Sea mercury inventory in the present study. Such sources may include produced water (water from the formation, produced along with the oil and gas, which can sometimes also contain the injection fluid and condensation water [105]) from offshore installations or cuttings piles (accumulation of drill cuttings that form during hydrocarbon drilling operations and consist of drilling fluid, subsurface rock debris and hydrocarbons [106]). Ongoing studies indicate that the bioavailable fraction of mercury from these piles is thought to be minimal. By contrast, more recent studies measuring mercury in North Sea seawater samples found concentrations around 0.5 ng l^-1^ [107], although others stated concentrations between 0.5 and 200 ng l ^-1^ [108]. The additional influx of mercury from offshore installations, while possible, is more likely to lead to concentration changes in the close vicinity of the platforms rather than on an ocean basin scale. Such limitations may, in part, be the cause for the need to use inflated environmental concentrations for the model calibration presented here. A further source of mercury to account for is decommissioning pipelines. Estimates provided for post-cleaning mercury concentrations assume that there is a uniform distribution of mercury along the length of the pipeline [32] and that the cleaning efficacy of 97% can be obtained throughout [109]. Past studies have highlighted that mercury accumulation is likely to increase the low points of pipelines, as well as areas of stagnation and changes in the flow regime, such as corners [110]. It was determined that post-cleaning, 7.7% of the remaining mercury were stable mercury salts and 92.3% were Hg^0^ [109]. Although leaching tests run with these samples indicated no increase in seawater mercury concentration over 112 days [111], it is not known how steel corrosion and marine microbes may influence such behaviour. Additionally, various parameters are known to influence the speciation of mercury within the pipeline, as highlighted in Kho *et al*. [18], which may further influence the risk posed to the local food web. This work has highlighted the need to improve our understanding of the actual mercury inventory in the North Sea and other ocean basins.

The previously discussed lack of data also meant that concentrations in some of the species modelled here had to be approximated from species of the same trophic level, but not necessarily with the same behaviour. This was the case for the plankton communities, as no data could be found for North Sea species. Thus, the uptake rates calculated here, and in turn the bioaccumulation, of mercury into the North Sea model was based on phytoplankton communities in the southern Baltic Sea [66]. This may account for a large fraction of the discrepancy between the modelled and observed mercury concentration in higher trophic-level species.

Lastly, a better understanding of the mobility and bioavailability of different mercury species, marine mercury methylation [19] and the mercury inventory in subsea infrastructure [15] is needed. The chemical speciation may be modelled by, for example, PHREEQc [112], a programme for geochemical calculations for one-dimensional transport, speciation and batch reactions. However, here too, the output is limited by our knowledge of speciation behaviour and influencing characters, as well as the reliability of input data. In addition to chemical speciation modelling, biological speciation (or methylation) needs to be taken into consideration. Past studies worked with concentration values for methylmercury only, examining the potential impact of changing climates on the uptake of methylmercury [53]. A study aiming to determine the methylation rate and potential of marine ecosystems highlighted that while certain parameters can be considered globally important, this biological speciation was a highly localized event and could not be predicted with current tools [113]. In addition, the inclusion of additional environmental compartments to represent the sediment and microbial community would improve the modelling of mercury speciation and availability in EwE.

### 4.4. Further considerations

Government statistics indicate that 80% of the seafood consumed in the UK consists of cod, haddock, salmon, tuna and various prawn species [114]. Such preferences were not considered in the present work, but future research would benefit from a more detailed assessment of society’s seafood consumption habits. This is especially relevant when considering contaminants such as methylmercury, with the potential to bioaccumulate and biomagnify, as all preferred fish species fall within trophic levels ≥4 and thus pose an increased risk of exceeding the FS for mercury, as well as the EWI_MeHg_ [115]. It should also be noted that most of the salmon consumed in the UK is farmed, thus being fed with fishmeal containing marine caught species of a lower commercial value such as shrimp or sand eel. A study conducted on Canadian farmed versus wild-caught salmon determined a negligible difference in mercury concentration in both salmon types but did determine a higher bioaccumulation factor for farmed individuals [116]. Although observing a similar trend in a larger database study for mercury in seafood, it was noted that farmed fish were understudied and that large discrepancies existed between the database estimated mean mercury concentration and the US Food and Drug Administration mercury monitoring programme estimates for most seafood examined in the study [117]. Future studies could also assess the impact of fishmeal diets on mercury accumulation in farmed fish in more detail to determine the potential health benefits or risks. These insights, and the bioaccumulative and biomagnifying nature of methylmercury, mean that a potential consequence of a future increase in seafood contamination is unavoidable and may be a reassessment of the dietary recommendations for seafood consumption. While reducing or eliminating seafood consumption may initially address the risk of increased methylmercury exposure, this may lead to consequences for both human health and the environment [118]. This highlights that there are many factors highlighting the importance of monitoring contaminant levels in marine food webs.

## 5. Conclusion

With decommissioning being in its infancy, all current proposals and conducted projects are executed in accordance with best practices and current scientific knowledge [119]. The present study, along with previous works [15,16,19], has highlighted gaps in the understanding of how contaminants associated with offshore infrastructures behave in the marine environment over time. With mercury having been associated with offshore oil and gas installations, the resulting potential environmental release in the case of *in situ* decommissioning could be a serious threat to the ecosystem and human health [15,16,19]. We determined data gaps in the mercury inventory for the North Sea, which may also affect other oceans. A global effort to address such gaps, for all contaminants of concern to the marine environment, would significantly improve our understanding of the potential risks of offshore activities on the marine food web and, by extension, human health and the relevant industries. Additionally, research should focus on determining the bioavailable fraction of methylmercury in seafood and the stomach uptake, thus allowing for more accurate estimates and future risk predictions.

This study aimed to apply a previously developed method for mercury bioaccumulation modelling to the North Sea marine ecosystem in order to assess potential future economic and human health risks of mercury released into the North Sea. All findings indicate that although an increase in accumulated mercury could be assumed, no further human health implications are expected under present assumptions. Uncertainties remain in the current data on both environmental mercury inventory and influx, as well as the mercury behaviour in the marine environment and its uptake by and speciation within marine organisms. Bridging these gaps would provide a great benefit for both the industry and the scientific community. It would allow for a more robust risk assessment to be conducted for offshore decommissioning practices, ensuring that decisions are made on the most holistic assessment of each asset. Moreover, it would provide a basis for the assessment of other contaminants, as well as the cumulative impact of various contaminants, greatly improving our ability to conserve the world oceans and offer more targeted mitigation solutions for marine pollution. The development of a global database for contaminant concentrations in marine species would greatly improve the ability to determine potential economic implications for the future of the fisheries industry. Moreover, the human risk determination method presented here would benefit from being tested on larger datasets with more detailed insights into consumer habits, as well as mercury organ/tissue distribution and speciation in marine organisms, species-specific assimilation efficiency and the uptake availability and efficacy of methylmercury by humans from different fish species and food preparation methods [92].

## Data Availability

All used data has been included in supplementary materials and is freely accessible with the publication [120].
